# *Clostridium Butyricum 337279* shapes the gut microbiota to attenuate metabolic disorder in diet-induced obese mice

**DOI:** 10.3389/fmicb.2025.1580847

**Published:** 2025-05-09

**Authors:** Xuejiao Zhang, Zhiyu Li, Junjun Cao, Haipeng Sun, Wenyan Niu

**Affiliations:** Key Laboratory of Immune Microenvironment and Disease of the Educational Ministry of China, NHC Key Lab of Hormones and Development and Tianjin Key Lab of Metabolic Diseases,Tianjin Medical University Chu Hsien-I Memorial Hospital and Institute of Endocrinology, Tianjin, China

**Keywords:** gut microbiota, systemic inflammation, branched-chain amino acids, metabolic disorder, insulin resistance

## Abstract

**Aims:**

Obesity is one of the important challenges to public health worldwide. Dysbiotic intestinal microbiota is a key factor in the onset and progression of obesity and related diseases. Short chain fatty acids (SCFAs) derived from *butyricogenic bacteria* has beneficial effects on obesity. *Clostridium Butyricum 337279* (*C.B*), one of the SCFA producing bacteria, has been used to treat inflammatory bowel disease. The effect of *C.B* on obese mice remains unclear.

**Methods:**

A high fat diet (HFD)-induced mouse model of obesity was constructed, and the mice were treated with *C.B* to examine their role on obesity and related metabolic disorder. RT-qPCR, Western blotting, immunohistochemical staining, and 16S rRNA gene sequencing were performed to investigate the role and mechanism of *C.B*. Plasma levels of BCAA and BCKA were detected by Shimadzu LC-20 AD liquid chromatography (LC) system.

**Results:**

Here we demonstrated that oral administration of *C.B* effectively alleviated HFD-induced obesity and associated metabolic disorders, including glucose intolerance and hyperlipidemia, as well as systemic inflammation, as evidenced by reduced levels of LPS, TNF-*α*, and IL-1β. *C.B* alleviated intestinal flora imbalance and modulated the composition of gut microbiota and their metabolites in HFD-induced obese mice. It also significantly ameliorated intestinal barrier disorders by increasing protein level of tight junction proteins ZO-1 and occludin. Importantly, dietary *C.B* potentially suppressed bacterial biosynthesis of branched-chain amino acids (BCAA) and reduced the excessive accumulation of BCAA in plasma, suggesting its role in restoring BCAA metabolism of mice.

**Conclusion:**

*C.B* intervention significantly ameliorated gut microbiota imbalance in obese mice and alleviated obesity-related metabolic disorders by upregulating the expression of tight junction proteins (ZO-1 and occludin), attenuating endotoxemia and systemic inflammation and reducing microbial-derived BCAA production.

## Graphical abstract

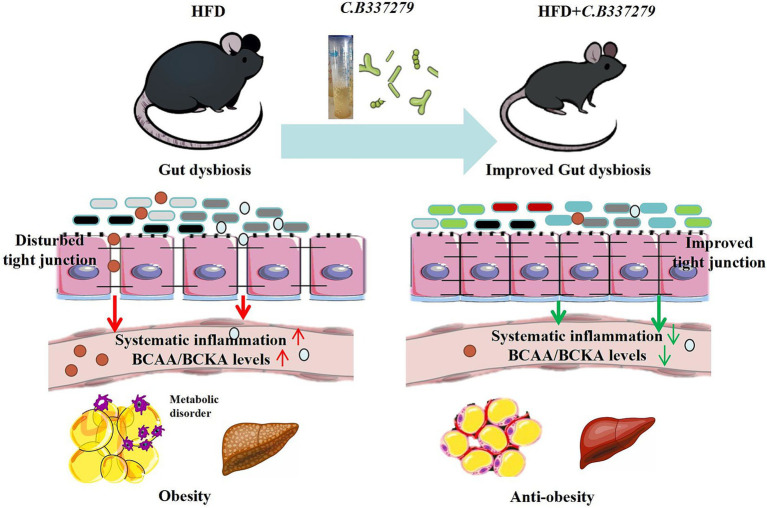

## Introduction

Metabolic syndrome (MetS), a clinical constellation encompassing obesity, hyperlipidemia, insulin resistance (IR), and metabolic dysfunction-associated steatotic liver disease (MASLD) (Lemieux and Després, 2020), is intricately linked to gut microbial dysbiosis (Dabke et al., 2019). Emerging evidence highlights microbial metabolites-particularly short-chain fatty acids (SCFAs; acetate, propionate, and butyrate)-as pivotal mediators of host-microbiome crosstalk that modulate obesity progression (Mann et al., 2024). SCFAs are predominantly produced by C*lostridium* species. They are primarily absorbed by intestinal epithelial cells, and directly activate G-coupled-receptors, inhibit histone deacetylases, and serve as energy substrates (Wong et al., 2006; Cresci et al., 2017; Koh et al., 2016). Beyond their role in mucosal protection, SCFAs regulate systemic energy homeostasis and mitigate metabolic inflammation (Yao et al., 2022). Notably, HFD-induced intestinal barrier dysfunction contributes to the development of metabolic disorders and colitis, both of which can be effectively alleviated by prebiotic interventions (Kang et al., 2022; Lee et al., 2022). Orally administered probiotic *Clostridium butyricum* produces large amounts of SCFAs in intestinal tracts (Cassir et al., 2016). However, the potential therapeutic effects of *Clostridium butyricum* on metabolic syndrome remain unclear, and its application as a therapeutic intervention faces significant challenges.

The intestinal barrier of obese mice is compromised, allowing microbiota-derived metabolites to translocate from the gut lumen into systemic circulation (Riedel et al., 2021). These microbial metabolites subsequently infiltrate key metabolic organs, including the liver and adipose tissue, thereby inducing systemic inflammation and contributing to the pathogenesis of MetS (Tilg et al., 2020; Winer et al., 2016). Extensive research has demonstrated that HFD-induced gut microbiota dysbiosis (GMD) generates pathogenic metabolites and compromises intestinal barrier integrity, ultimately resulting in enhanced intestinal permeability. This may facilitate toxin translocation into the systemic circulation to induce metabolic diseases. Studies have revealed the relationship between dietary BCAA and gut microbiota (Karusheva et al., 2019; Wang et al., 2023; Li et al., 2022). BCAA (valine, leucine, isoleucine) are essential amino acids. Elevated BCAAs levels are associated with metabolic diseases such as obesity and diabetes (Zhou et al., 2019), which are often accompanied by microbiome disorders. Evidence showed that gut microbiota play a role in BCAA metabolism (Ridaura et al., 2013; Pedersen et al., 2016; Oh et al., 2021). Several gut microbiome were identified as the most abundant amino acid biosynthesis bacteria (Neis et al., 2015). Given the well-established association between disrupted gut microbiota and obesity (Mitev and Taleski, 2019), the potential relationship between GDM and elevated BCAA levels, remains to be elucidated.

Here we showed that *Clostridium butyricum 337279* (*C.B 337279*), a butyrate-producing strain, counteracts HFD-induced gut dysbiosis, intestinal hyperpermeability, and metabolic inflammation. Concurrently, it restored gut barrier integrity via upregulation of tight junction proteins (ZO-1, occludin), reducing systemic endotoxemia and proinflammatory cytokines (IL-6, TNF-*α*). Metagenomic analysis revealed that *C.B 337279* selectively suppressed BCAA-producing pathobionts (*Proteobacteria*, *Desulfovibrionaceae*). This microbial shift correlated with decreased plasma levels of BCAAs and BCKAs, which are established drivers of IR. These results establish *C.B 337279* as a microbiota-modulating therapeutic agent for metabolic disorders and redefine BCAA homeostasis as a mechanistic target for microbiome-based interventions.

## Methods

### *In vitro C.B 337279* culture

*Clostridium Butyricum 337279* (BNCC *337279*) was purchased from the Beijing Beina Innovation Alliance Institute of Biotechnology (BNCC). *C.B 337279* was cultured in Reinforced Clostridial Medium (RCM) that contained 10 g/L peptone, 5 g/L yeast extract, 3 g/L glucose, 5 g/L NaCl, and 1 g/L soluble starch, adjusted to pH 6.8. *C.B 337279* was cultured at 37°C for 48 h and the bacterial suspension was centrifuged at 8000 rpm for 20 min, the cultured bacterial cells were resuspended in normal saline for gavage. Oral administration of *C.B* (1–5 × 10^8^ CFU for each mouse) was performed every other day for 8 weeks.

### Animals and treatment

**Figure 1:**
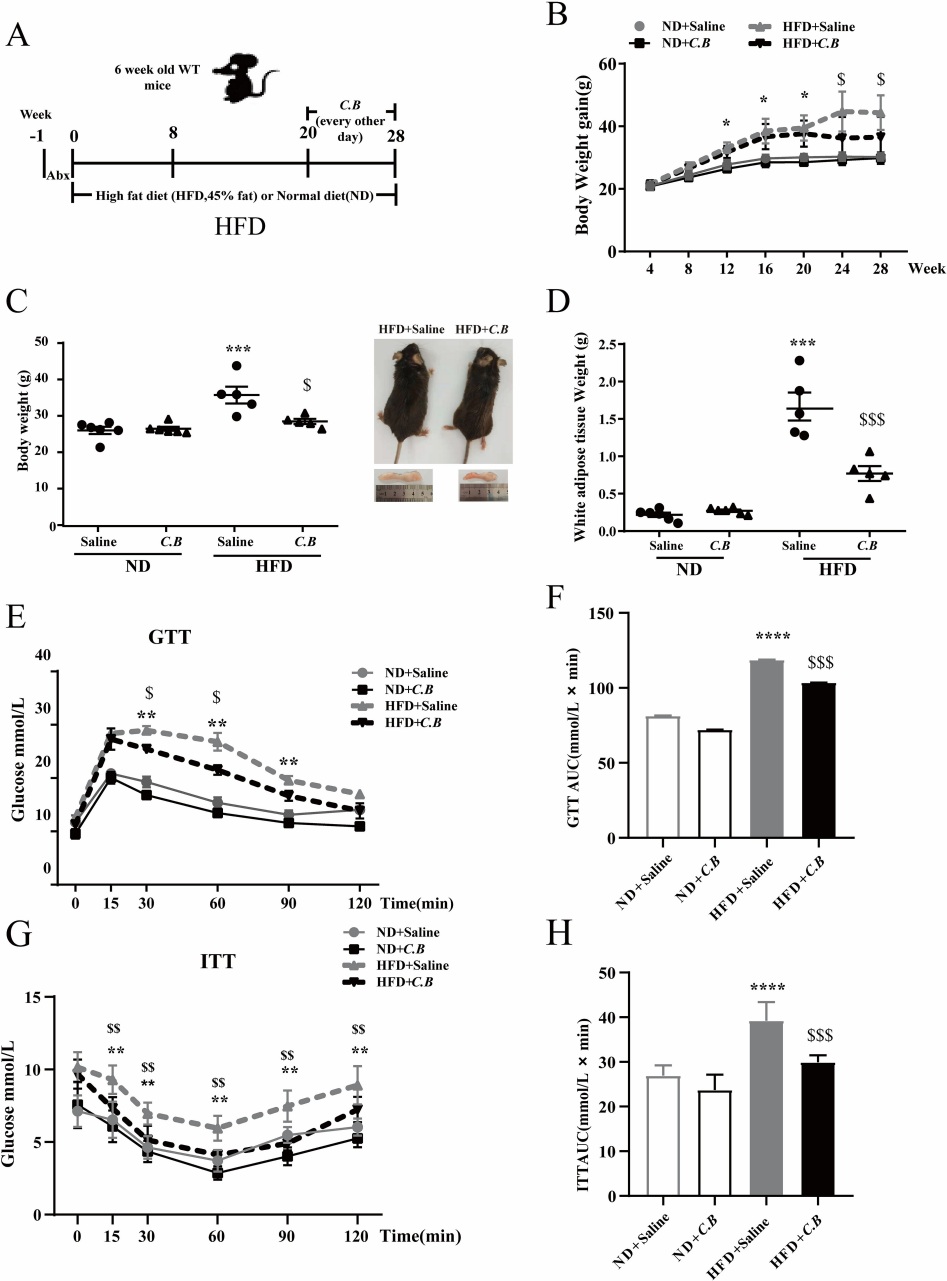
Dietary *C.B* alleviated IR in obese mice. **(A)** Schematic diagram showing the study design for *C.B* administration in HFD mice. **(B)** Time series of body weights. **(C)** Body weight and representative mice from the HFD and HFD + *C.B* groups. **(D)** The weight of WAT from different group. **(E,F)** Effect of *C.B* on glucose tolerance measured by oral glucose tolerance test (OGTT). Right: Area under the curve (AUC). **(G,H)** Effect of *C.B* on percentage of initial blood glucose level during insulin tolerance test (ITT). Right: AUC. Statistical significance was determined by two-way ANOVA with Tukey tests for multiple-group comparisons, (*n* = 5–7). * *p* < 0.05, ** *p* < 0.01 vs. ND + Saline, and ^$^ *p* < 0.05, ^$$^ *p* < 0.01 vs. HFD + Saline.

Six-week-old wild type C57BL/6 male mice were purchased from Huafukang Institute of Experimental Animal Research, Beijing, China (Table 1). After 7 days antibioticmixture (Abx) treatment, mice were randomly divided into two groups fed normal control diet (ND) or 60% HFD for 20 weeks, respectively. Each group mice were further divided into saline groups and *C.B* groups (ND + Saline, ND + *C.B*, HFD + Saline, HFD + *C.B*) for the last 8 weeks (Figure 1A). Following an 8-week intervention period, mice were fasted for 6 h, anesthetized with 3% isoflurane, and euthanized via cervical dislocation. Content and mucosa of the small intestine, cecum and colon were collected following immediately frozen, and stored at −80°C. All animal procedures were approved by the Animal Care and Use Committee of Tianjin Medical University and were conducted in accordance with the Guide for the Care and Use of Laboratory.

**Table 1:** Diets used in this study.

Product #	D12450B (10%fat)		D12451 (45%fat)	
%	gm	*kcal*	gm	*kcal*
Protein	19	20	24	20
Carbohydrate	67	70	41	35
Fat	4	10	24	45
Total		100		
kcal/gm	3.8		4.7	

Ingredient	gm	*kcal*	gm	*kcal*
Casein	200	800	200	800
L-Cystine	3	12	3	12
Corn starch	315	1,260	72.8	291
Maltodextrin 10	35	140	100	400
Sucrose	350	1,400	172.8	691
Cellulose, Bw200	50	0	50	0
Soybean oil	25	225	25	225
Lard	20	180	177.5	1,598
Mineral MixS10026	10	0	10	0
Dicalcium phosphate	13	0	13	0
Calcium carbonate	5.5	0	5.5	0
Potassium citrate.1 H20	16.5	0	16.5	0
Vitamin MixV10001	10	40	10	40
Choline bitartrate	2	0	2	0
FD&C Yellow Dye #5	0.05	0	0	0
FD&C Red Dye #40	0	0	0.05	0
FD&C Blue Dye #1	0	0	0	0
**Total**	**1055.1**	**4,057**	**858.15**	**4,057**

### Measurements of metabolic indices and serum cytokines

Liver tissues (30 mg) were homogenized in ice-cold chloroform:methanol extraction buffer (2:1 v/v; Sigma-Aldrich C2432/M1775). Following 16-h solvent extraction at 4°C, samples were phase-separated by adding 300 μL deionized water and centrifuged at 12,000 × g for 10 min (4°C). Lipid-containing organic phases were vacuum-dried (CentriVap Concentrator, Labconco) and reconstituted in 200 μL 5% Triton X-100 (Sigma-Aldrich T9284). Hepatic triglyceride (TG) and total cholesterol (TC) levels were quantified using enzymatic assay kits (Zhongsheng Beikong, #ZS-1011-TG/#ZS-1012-TC). The serum cytokines including IL-1β, IL-6, lipopolysaccharide-binding protein (LBP), lipopolysaccharide (LBS) and TNFα were detected with commercial kits according to the manufacturer’s introduction.

### Oil Red O staining, histological analyses and immunohistochemistry

Fresh liver specimens were embedded in optimal cutting temperature compound (OCT; Sakura #4583) and snap-frozen in liquid nitrogen-cooled isopentane. Cryoblocks were sectioned at 7 μm thickness using a cryostat (Leica CM1950), and fixed in 10% neutral buffered formalin for 10 min. Sections were deparaffinized and rehydrated following with Hematoxylin and eosin staining or incubating with anti-ZO-1(D6L1E) antibody (Cell Signaling Technology, #13663), anti-Occludin-1(E6B4R) antibody (Cell Signaling Technology, #91131) at 4°C for overnight, respectively. Then the sections were incubated with secondary antibody for 30 min. Images were acquired under a Leica microscope.

### RNA extraction and real-time PCR

Total RNA from frozen livers or white adipose tissue were isolated with TRlzol reagent (15596026, Invitrogen) and subjected to complementary DNA synthesis by reverse-transcription with random primers. The mRNA expression of different genes was normalized to that of *β*-actin. The quantitative PCR procedure followed the Minimum Information for Publication of Quantitative Real-Time Polymerase Chain Reaction Experiments guidelines. Sequences of primers for quantitative PCR are shown in Table 2.

**Table 2:** List of the gene-specific primer sequences used in this study.

Gene	Species	Stand	Sequence
*β-actin*	Mouse	Forward primer	CCTCTATGCCAACACAGTGC
		Reverse primer	CCTGCTTGCTGATCCACATC
*cd36*	Mouse	Forward primer	TGGTCAAGCCAGCTAGAAA
		Reverse primer	CCCAGTCTCATTTAGCCAC
*IL-1β*	Mouse	Forward primer	GCCCATCCTCTGTGACTCAT
		Reverse primer	AGGCCACAGGTATTTTGTCG
*tnf-*α	Mouse	Forward primer	CCAGACCCTCACACTCAGATC
		Reverse primer	CACTTGGTGGTTTGCTACGAC
*Mcp-1*	Mouse	Forward primer	TGGCTCAGCCAGATGCAGT
		Reverse primer	CCAGCCTACTCATTGGGATCA

### 16S rRNA analysis

Total genomic DNA was extracted from the digestive tract of the colon using the QIAamp DNA Stool Mini Kit (Qiagen GmbH, Hilden, Germany) according to the manufacturer’s instructions. The concentration of genomic DNA were measured and purified. The integrity of extracted genomic DNA was determined by electrophoresis on 1% (w/v) agarose gel. Primer sequencing and bioinformatics analysis were conducted by Novogene (Beijing, China) using paired end sequencing on the Illumina HiSeq platform. The V3–V4 region of the bacterial 16S rRNA gene was amplified by PCR using bacterial universal primers to fully define bacterial composition and abundance. Use Uparse (Uparse v7.0.1001) to cluster the obtained sequences into operational taxonomic units (OTUs) with 97% sequence identity for *α* diversity and *β* diversity analysis.

### BCAA measurement

The plasma levels of BCAA and BCKA were detected by multiple reaction monitoring (MRM) in positive and negative electrospray ionization mode, respectively. Chromatographic separation was achieved on an Agilent ZORBAX SB-C18 (150 × 3 mm, 5 μm) column, and temperature controlled at 50°C. The standards and samples were separated using a mobile phase consisting of methanol/water (20:80, v/v) with 0.1% formic acid (eluent A) and acetonitrile (eluent B). The mobile was 0% B initially, which held for 1.5 min and then increased to 90% over 0.5 min. The mobile phase was held at 90% B for 5 min and then re-equilibrated to 0% B and held for 6 min. The flow rate was 0.9 mL/min. An aliquot of 10 μL plasma was spiked with 110 μL methanol/acetonitrile/water (50:50:10, v/v/v) containing stable-isotope-labeled internal standards (48 ng [D3] Leucine, 24 ng [13C4, D3] KIV sodium salt, and 16 ng [D3] KIC sodium salt) and remained on ice for 10 min before being centrifuged at 14000 g at 4°C for 10 min. The supernatant was collected and dried with a stream of nitrogen. Following this, the samples were reconstituted in 100 μL of methanol/water (20:80, v/v) for analysis. The injection volume was 10 μL. Data acquisition and quantitation were performed with Analyst 1.7 and MultiQuant 3.0 software, respectively.

### Glucose and insulin tolerance tests

For the glucose tolerance test (GTT), mice were fasted for 9 h and subsequently administered an intraperitoneal injection of glucose (2 g/kg body weight). Blood glucose levels were measured using a handheld glucometer (Accu-CHEK; Roche, Mannheim, Germany) from tail vein blood samples collected at 0, 15, 30, 60, 90, and 120 min. For the insulin tolerance test (ITT), mice fasted for 9 h received an intraperitoneal bolus injection of insulin (1.5 U/kg body weight). Glucose was measured as described above.

### Statistical analysis

Statistical analyses were performed using GraphPad Prism 8.0.1.^1^ Significant differences were evaluated using two-way analysis of variance (ANOVA) with Tukey tests for multiple-group comparisons. All values are expressed as mean ± SEM. *p* < 0.05 was considered significant.

## Results

### *C.B* attenuated HFD-induced obesity and alleviated obesity associated-IR

We initially investigated whether *C.B* exerts any effects on MetS in mice with HFD-induced obesity. Mice fed with HFD for 20 weeks showed significant increases in body weight, white adipose tissue weight, and impaired glucose tolerance test compared to mice fed with chow diet (Figures 1B–F). Notably, oral administration of *C.B* (1–5 × 10^8^ CFU) administered every other day for 8 weeks effectively attenuated HFD-induced body weight gain (Figures 1B,C). This effect was not associated with food intake (Supplementary Figure S1). Liver weight was not changed (data is not shown), while the weight of white adipose tissue was significantly reduced by *C.B* (Figure 1D). In addition, *C.B* significantly improved glucose tolerance (Figure 1E) and insulin sensitivity (Figure 1G). Furthermore, *C.B* administration significantly reduced the area under the curve (AUC) of GTT (Figure 1F, *p* < 0.05) and improved insulin tolerance, as evidenced by the percentage change in ITT at both 30- and 60-min time points (Figure 1H, *p* < 0.05). These findings collectively demonstrate that *C.B* treatment not only attenuated body weight gain but also enhanced insulin sensitivity in diet-induced obese mice.

### *C.B* ameliorated HFD-induced hyperlipemia and ectopic lipid deposition

**Figure 2:**
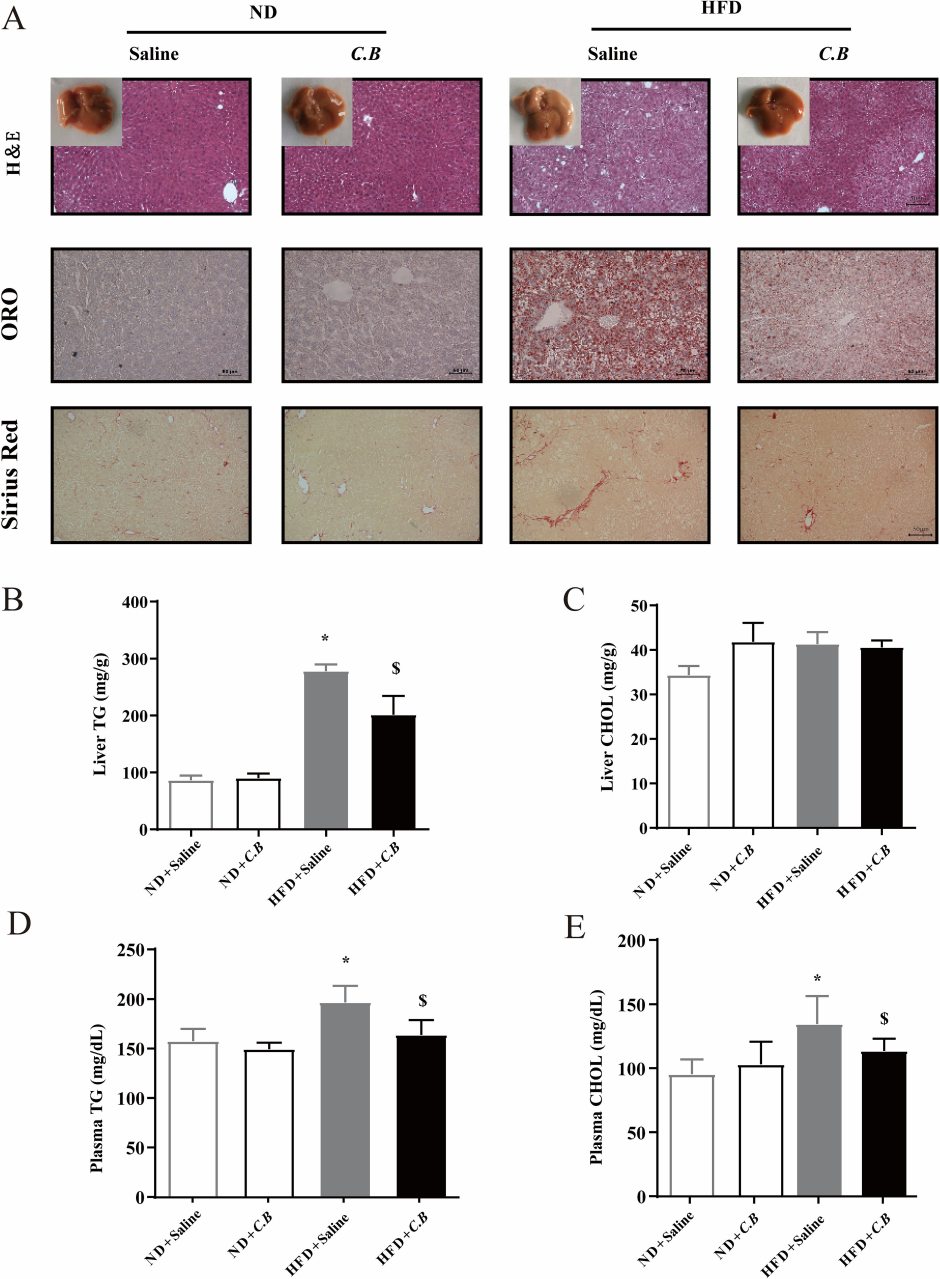
*C.B* ameliorated hyperlipemia and ectopic lipid deposition of obese mice. **(A)** Representative images of liver sections stained with H&E, Oil Red O, and Sirius Red (scale bars, 50 μm). **(B,C)** Quantification of hepatic triglycerides and cholesterol. **(D,E)** Serum levels of triglycerol and cholesterol. Statistical significance was determined by two-way ANOVA with Tukey tests for multiple-group comparisons, data are expressed as mean ± SEM, (*n* = 5–7). * *p* < 0.05 vs. ND + Saline, ^$^ *p* < 0.05 vs. HFD + Saline.

Prolonged HFD feeding has been consistently associated with progressive hepatic alterations, including steatosis, fibrosis, and inflammatory responses. To investigate the therapeutic potential of *C.B* intervention, we subsequently evaluated its effects on the progression of MASLD in the HFD-induced mouse model. As shown in Figure 2A, *C.B* treatment significantly ameliorated hepatic lipid accumulation and fibrogenesis compared to the HFD-fed control group. Consistent with the histological observations, quantitative analysis demonstrated a substantial reduction in hepatic lipid content, with triglyceride (TG) levels showing a particularly significant decrease of 33.3% (*p* < 0.05) in *C.B*-treated mice relative to HFD controls (Figures 2B,C). Furthermore, *C.B* administration led to a significant improvement in serum lipid profiles, as indicated by pronounced reductions in both TG and total cholesterol (CHOL) levels (Figures 2D,E). Taken together, these findings demonstrate that *C.B* treatment effectively ameliorates systemic lipid metabolic disorders, particularly in both circulating and hepatic lipid homeostasis.

### *C.B* reduced systemic inflammatory in obese mice

**Figure 3:**
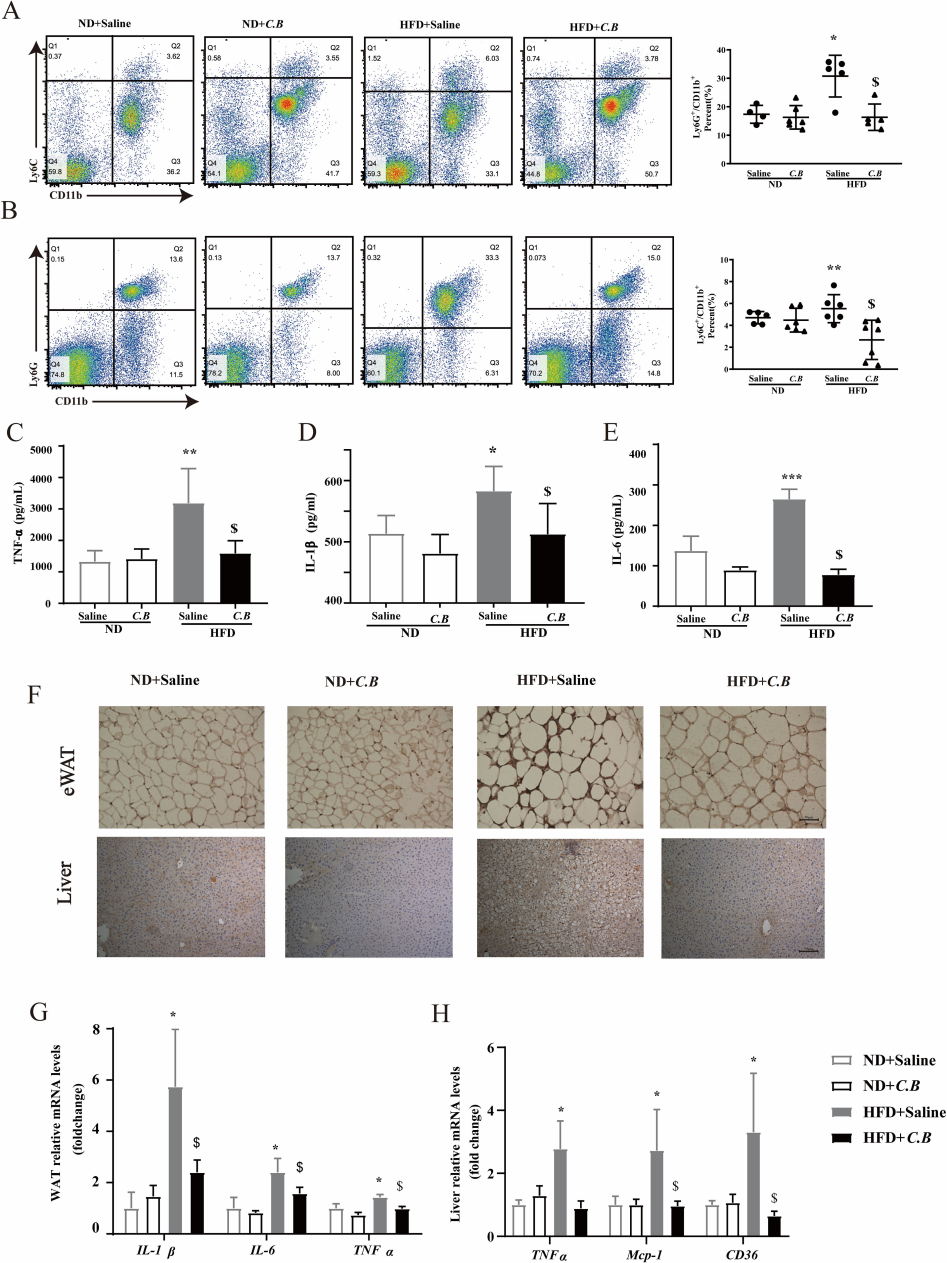
*C.B* protects systemic inflammation of obese mice. **(A)** Fluorescence-activated cell sorting (FACS) analysis of CD11b^+^Ly6C^hi^Ly6G^−^ in PBMC. **(B)** FACS analysis of CD11b^+^Ly6C^hi^Ly6G^+^. **(C–E)** Serum levels of IL-6, IL-1b and TNF-ɑ were detected by ELISA. **(F)** Representative images for immunostaining of F4/80 in adipose tissues and liver section from four groups (scale bars, 50 μm). **(G,H)** qPCR results of pro-inflammatory genes expression in adipose and liver tissue from four groups. Statistical significance was determined by two-way ANOVA with Tukey tests for multiple-group comparisons. Data are mean ± SEM, (*n* = 5–7). * *p* < 0.05 vs. ND + Saline, ^$^ *p* < 0.05 vs. HFD + Saline.

SCFAs have been well-documented to exert immunomodulatory effects on systemic inflammatory responses. Based on this established mechanism, we subsequently investigated whether *C.B* administration could ameliorate metabolic dysfunction through the attenuation of systemic inflammation. The immunophenotypic characterization of peripheral blood mononuclear cell (PBMC) subpopulations was performed using flow cytometry. As shown in Figure 3A, the proportion of CD11b^+^Ly6C^hi^Ly6G^−^ cells in HFD group was higher than that in ND group (3.62% vs. 6.03%, *p* < 0.01). After *C.B* administration, the proportion of monocytic cells in HFD group reduced significantly, which was in accordance with the reduced proportion of CD11b^+^Ly6C^−^Ly6G^hi^ cells (Figure 3B). Consistent with the above findings, serum analysis demonstrated significantly elevated levels of pro-inflammatory cytokines, including TNF-*α*, IL-1β, and IL-6, in HFD-fed mice compared to ND controls (*p* < 0.01). Notably, oral administration of *C.B* effectively attenuated these inflammatory responses, as evidenced by significant reductions in cytokine levels (Figures 3C–E, *p* < 0.05). In line with these observations, *C.B* treatment significantly reduced the infiltration of F4/80^+^ macrophages in both hepatic and WAT compartments of HFD-fed mice (Figure 3F). Most notably, *C.B* administration markedly downregulated the expression of pro-inflammatory markers in HFD-fed mice, with particularly pronounced effects observed in WAT (*p* < 0.01, Figures 3G,H). Collectively, these findings demonstrate that *C.B* possesses potent anti-inflammatory properties that effectively ameliorate obesity-associated inflammation in multiple metabolic tissues.

### *C.B* reduced HFD-induced intestinal permeability

**Figure 4:**
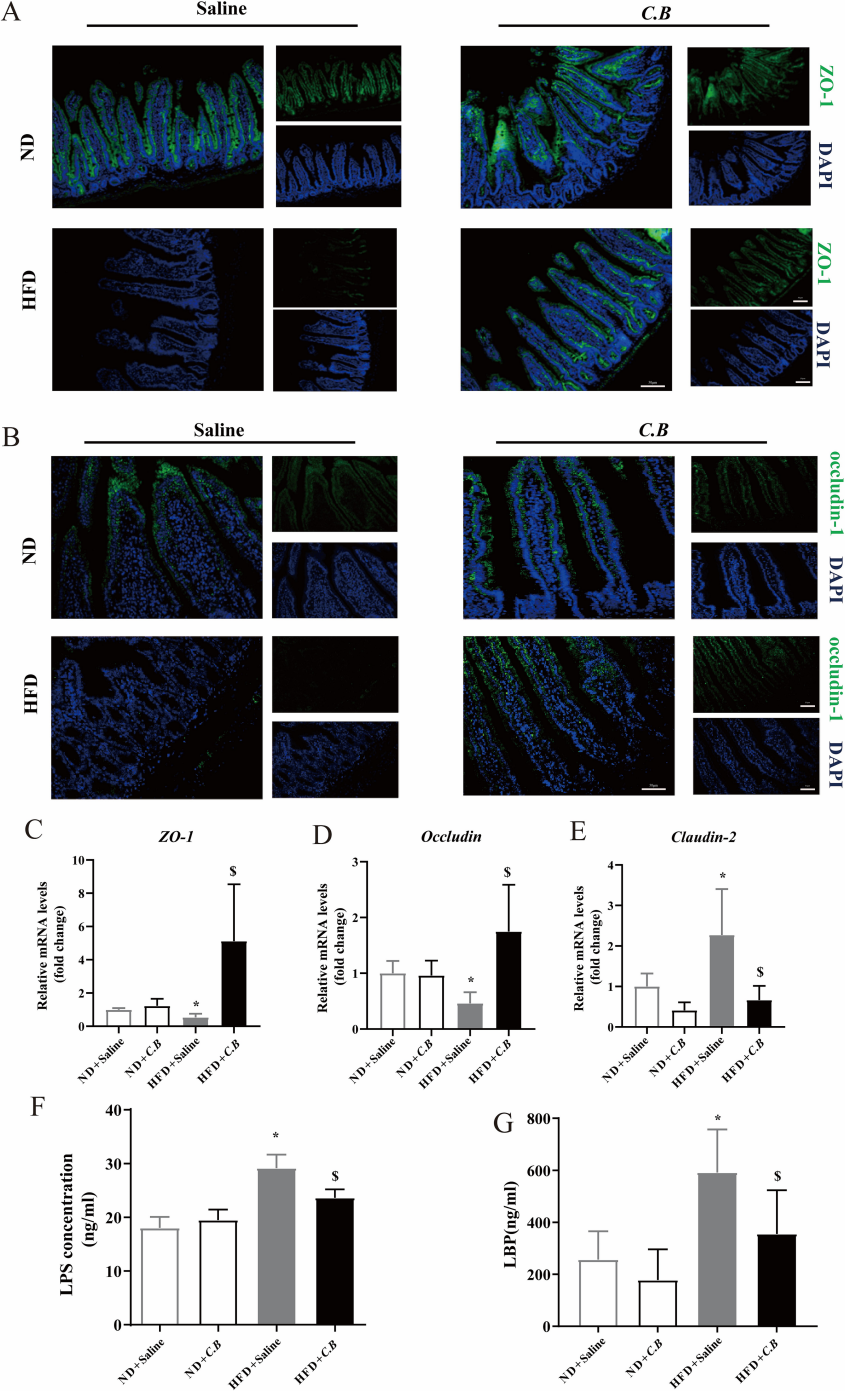
*C.B* protects the disturb of intestinal barrier in obese mice. **(A,B)** Immunohistochemistry of ZO-1 and occludin, (scale bars, 50 μm). **(C–E)** The expression of the zo-1, Occludin and Claudin-2 genes in the intestinal. **(F,G)** Serum LPS and LBP concentration. Statistical significance was determined by two-way ANOVA with Tukey tests for multiple-group comparisons. Results were shown as mean ± SEM (*n* = 6). ^*^ *p* < 0.05 vs. ND + Saline group, ^$^ *p* < 0.05 vs. HFD + Saline group.

A growing body of works suggests that early changes in the gastrointestinal (GI) barrier may contribute to both local, within the GI lining, and systemic inflammation in obesity (Acciarino et al., 2024; Nascimento et al., 2021). We next examined the effects of *C.B* on intestinal integrity. As the immunofluorescence data shown in Figures 4A,B, the tight junction proteins zonula occludens-1 (ZO-1) and occludin in HFD group were decreased, which was significantly reversed after *C.B* treatment. We also evaluated the integrity of the intestinal barrier. The mRNA levels of tight junction-related (TJ) gens in the intestinal tissue, including ZO-1 and occludin were higher in HFD + *C.B* group than that in HFD group (Figures 4C,D, *p* < 0.01). In comparison, claudin-2 in HFD + *C.B* group was lower than that in HFD group (Figure 4E, *p* < 0.05). In a line with these results, *C.B* treatment significantly decreased the circulation levels of LBP and LBS caused by HFD (Figures 4F,G), indicating a potential role of *C.B* in modulating intestinal permeability, which may contribute to the alleviation of systemic inflammation.

### *C.B* regulates the microbiota composition of the HFD fed mice

**Figure 5:**
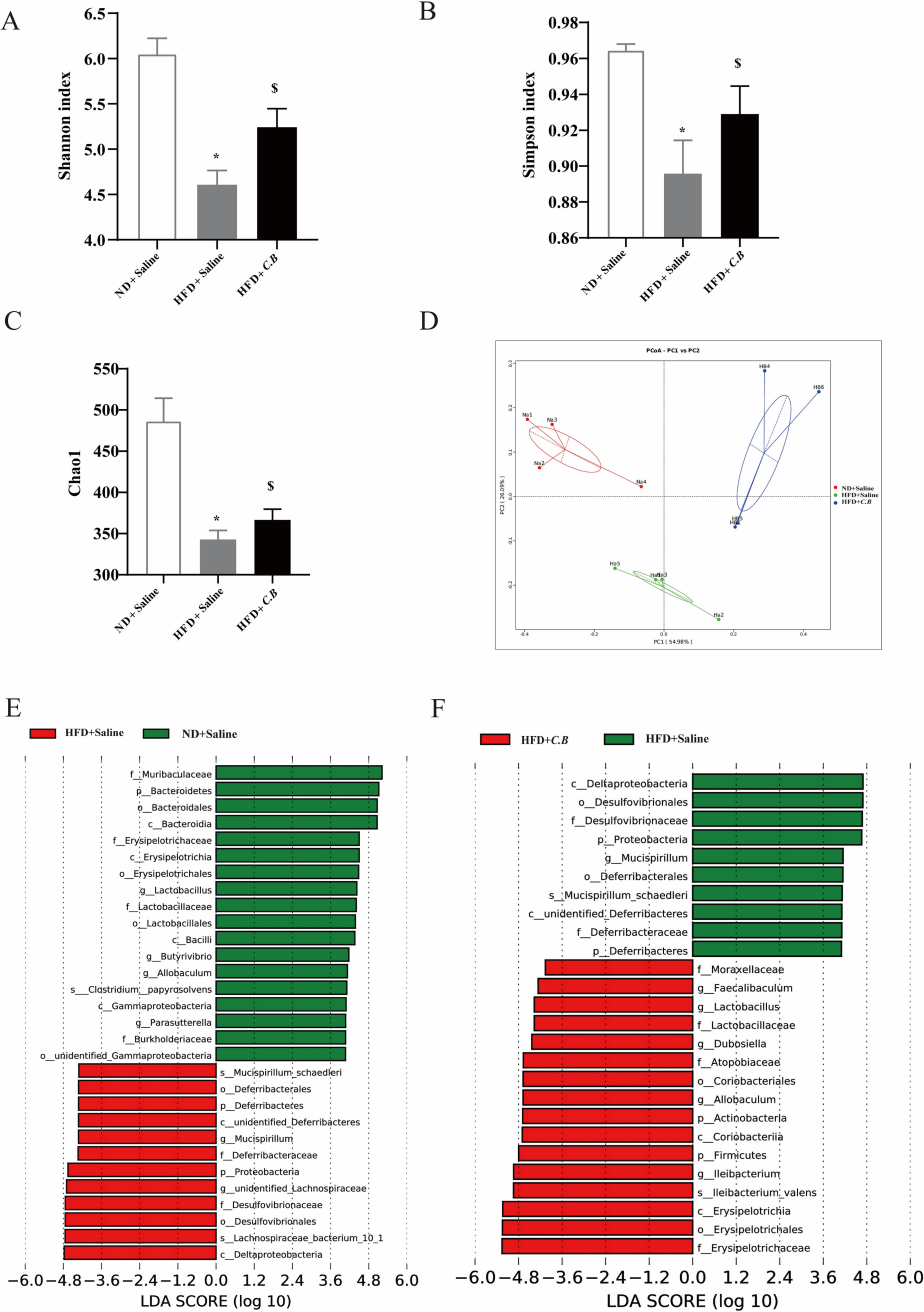
*C.B* protects gut microbiota dysbiosis in obese mice. **(A–C)** Alpha diversity between the chow and HFD groups: Shannon index **(A)**, Simpson index **(B)** and Chao1 index **(C)**. Statistical significance was determined by two-way ANOVA with Tukey tests for multiple-group comparisons. **(D)** The *β*-diversity of gut microbiota was determined by principal coordinate analysis based on the unweighted unique fraction metric distance. **(E,F)** LEfSe analysis shows the species that best explain differences between **(E)** the ND and HFD groups and **(F)** the HFD and HFD + *C.B* groups. The taxonomic levels were indicated as “s” = species; “g” = genus; “f” = family; “o” = order. LDA, linear discriminant analysis; PCoA, principal coordinates analysis. Results were shown as mean ± SEM (*n* = 4). ^*^ *p* < 0.05 vs. ND + Saline group, ^$^ *p* < 0.05 vs. HFD + Saline group.

The above data showed that *C.B* is crucial for mediating the intestinal permeability. We speculated that *C.B* may act as a gut microbiota-modulator to inhibit inflammation and improve dyslipidemia via remodeling the microbiota. To elucidate the impact of dietary *C.B* intervention on gut microbial ecology, we performed comprehensive 16S rRNA gene sequencing analysis to characterize alterations in intestinal microbiota composition. Microbial *α*-diversity was quantitatively assessed using established ecological indices, including observed operational taxonomic units (OTUs), Chao1 richness estimator, Shannon diversity index, and Simpson diversity index, across three experimental groups (ND + Saline, HFD + Saline, HFD + *C.B*). As illustrated in Figures 5A–C, Shannon’s diversity index was lower in the HFD group than ND group. This was restored by *C.B*. Moreover, the Simpson index and Chao1 index showed the same trend among three groups. Principal coordinates analysis (PCoA) of the fecal microbiota based on weighted_unifrac was used to measure overall structure difference among different groups. As demonstrated in Figure 5D, distinct clustering patterns were observed, indicating differential microbial community structures in response to *C.B* interventions. These results revealed that HFD feeding significantly reduced gut microbial richness, as evidenced by decreased observed species counts, while *C.B* intervention effectively restored bacterial diversity and induced structural reorganization of the gut microbiota community.

To identify differentially abundant microbial taxa across experimental groups, we performed linear discriminant analysis effect size (LEfSe) analysis. As illustrated in Figures 5E,F, the analysis revealed distinct microbial signatures with significant differences in taxonomic abundance spanning multiple phylogenetic levels, from phylum to species. LEfSe analysis identified several significantly enriched bacterial taxa in the HFD group, including *S_Mucispirillum_schaedleri* (species), *o_Deferribacterales* (order), *p_Deferribacteres* (phylum), *g_Mucispirillum* (genus), *f_Deferribacteraceae* (family), *p_Proteobacteria* (phylum), *f_Desulfovibrionaceae* (family), and *o_Desulfovibrionales* (order). Notably, comparative analysis revealed distinct microbial signatures in the *C.B*-treated group, demonstrating significant alterations in dominant bacterial populations compared to HFD controls. These findings suggest that *C.B* intervention induces substantial remodeling of the gut microbial community structure in HFD-fed mice.

### *C.B* act as a gut microbiota-modulator keeping the balance between dominant and inferior flora

**Figure 6:**
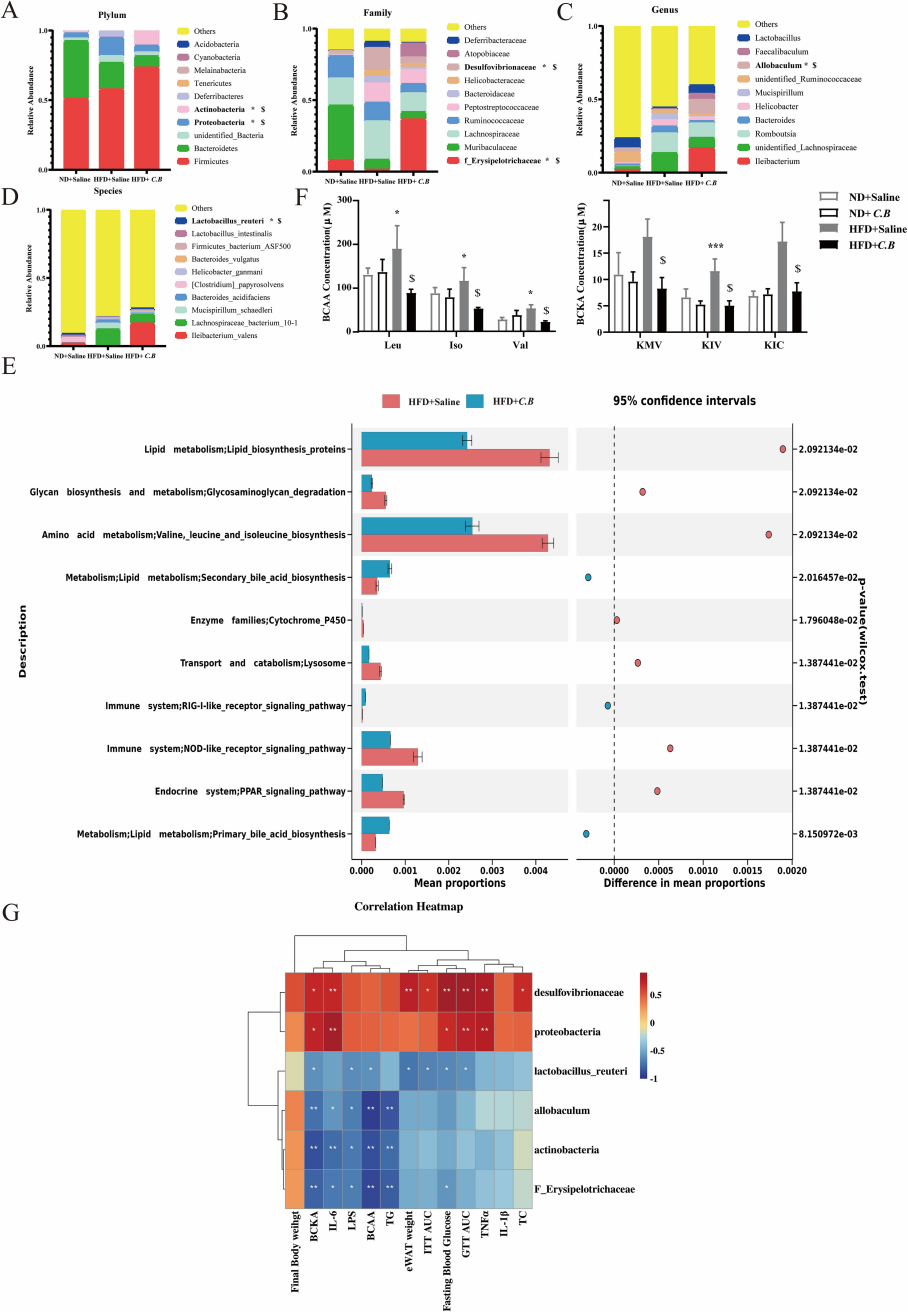
*C.B* act as a gut microbiota-modulator remodeling the abundance of flora related with BCAA biosynthesis and systematic inflammation. **(A–D)** Relative abundance of the gut microbiota at the plylums **(A)**, family **(B)**, genus **(C)**, and specie level **(D)**. Results were shown as mean ± SEM (*n* = 4).^*^ *p* < 0.05 vs. ND + Saline group, ^$^ *p* < 0.05 vs. HFD + Saline group. **(E)** Function prediction of the functional gene composition between the HFD group and HFD + *C.B* group based on Tax4Fun. The middle shows the difference ratio of functional abundance within the 95% confidence interval. **(F)** Plasma levels of BCAAs and BCKAs (*n* = 8). **(G)** Partial Spearman’s correlation analyses between significant metabolites and gut microbiota. All values are expressed as the mean ± SEM (*n* = 4). * *p* < 0.05, *** *p* < 0.001 vs. ND + Saline group, ^$^ *p* < 0.05 vs. HFD + Saline group.

To further clarify the role of *C.B* as a gut microbiota-modulator, we compared the relative abundances of the predominant phyla and species in the three groups, especially taxa whose abundances were altered in response to *C.B* administration. At the phylum level, taxonomic profiling revealed a significant increase in the relative abundance of *Proteobacteria* and a concurrent decrease in *Actinobacteria* in HFD-fed mice compared to ND controls. Remarkably, *C.B* intervention effectively normalized these HFD-induced phylum-level alterations, restoring the relative abundances of both *Proteobacteria* and *Actinobacteria* to levels comparable to those observed in ND mice (Figure 6A). At the family level, microbial analysis revealed that HFD-fed mice exhibited a significant increase in the abundance of *Desulfovibrionaceae*, a bacterial family associated with LPS production, coupled with a marked reduction in *Erysipelotrichaceae*, known for its protective role against hepatic steatosis. Importantly, *C.B* intervention effectively counteracted these HFD-induced alterations (Figure 6B), demonstrating its capacity to restore a beneficial microbial profile. Notably, at the genus level, *C.B* administration significantly enhanced the abundance of *Allobaculum*, a well-characterized butyrate-producing genus known for its role in SCFA biosynthesis and maintenance of gut epithelial integrity (Figure 6C). Furthermore, *C.B* intervention markedly increased the relative abundance of *Lactobacillus reuteri*, a well-established probiotic species with demonstrated beneficial effects on host metabolism and intestinal barrier function (Figure 6D).

Next, Tax4Fun2 was applied to predict functional profiles (KEGG pathways) from 16S rRNA data, and significant differences in metabolic pathways between the HFD and HFD + *C.B* groups were analyzed. Notably, cecal microbiota from HFD-fed mice demonstrated marked upregulation of key metabolic pathways, particularly those involved in lipid metabolism, carbohydrate processing, and glycan biosynthesis and metabolism (Figure 6E). Importantly, *C.B* administration demonstrated remarkable efficacy in modulating key metabolic processes, as evidenced by the significant downregulation of lipid biosynthesis pathways, BCAA biosynthesis, and systemic inflammatory signaling cascades (Figure 6E). It has been established that the circulating BCAA levels are associated with the risk of type 2 diabetes (Zhou et al., 2019; White et al., 2018). The serum metabolome of insulin-resistant individuals is characterized by increased levels of BCAA and BCKA (Wang et al., 2011). Surprisingly, we observed that *C.B* decreased the BCAA and BCKA levels of HFD-fed mice (Figure 6F).

### Correlation analysis of physical and chemical indicators with intestinal flora

To elucidate the potential functional relationships between the six most abundant OTUs and systemic metabolic parameters in HFD-fed mice, we performed comprehensive correlation analysis using Spearman’s rank correlation coefficient to assess associations with serum biochemical markers and obesity-related metabolic indices. *Proteobacteria* and *Desulfovibrionaceae* exhibited significant positive correlations with pro-inflammatory markers (TNF-ɑ and IL-6), glucose intolerance (GTT AUC), and branched-chain keto acid (BCKA) levels (Figure 6G). Furthermore, beneficial microbial taxa, including *Actinobacteria*, *Erysipelotrichaceae*, *Allobaculum*, and *Lactobacillus reuteri*, showed significant inverse relationships with key metabolic indicators, particularly the circulating BCAA/BCKA and LPS concentrations. These findings suggest that *C.B*-induced modulation of these bacterial populations may play a crucial role in ameliorating obesity-associated metabolic dysregulation by simultaneously regulating metabolite profiles of intestinal flora.

## Discussion

Accumulating evidence suggests that HFD-induced GMD represents a pivotal factor in the disruption of energy homeostasis, impairment of intestinal barrier integrity, and development of systemic low-grade inflammation (Fan et al., 2022; Ren et al., 2023; Potrykus et al., 2021). It is urgent to find strategies to alleviate the intestinal barrier and inflammation. Previous clinical studies have demonstrated that *Clostridium butyricum* effectively ameliorates dextran sulfate sodium (DSS)-induced intestinal pathologies, including fibrosis, inflammation, and barrier dysfunction in pediatric patients (Ma et al., 2022; Stoeva et al., 2021). However, the therapeutic potential of *Clostridium butyricum* in addressing obesity-associated intestinal complications remains largely unexplored. In this study, we demonstrated that *C.B 337279* effectively ameliorates HFD-induced metabolic disorders through gut microbiota remodeling and modulation of microbial metabolites in obese mice. Our findings reveal that administration of *C.B* during the final phase of HFD feeding significantly enhanced microbial *α*-diversity, as evidenced by increased Shannon and Simpson indices. Furthermore, PCoA of *β*-diversity revealed distinct clustering patterns and significant alterations in microbial community structure between *C.B*-treated and HFD control groups.

The gut microbiota plays a pivotal role in the pathogenesis of diet-associated metabolic disorders, with substantial evidence demonstrating its involvement in the development of intestinal barrier dysfunction (leaky gut syndrome) and obesity-related systemic metabolic complications (Wang et al., 2022; Xu et al., 2022). Consistent with previous findings, our analysis revealed that HFD decreased the richness and diversity of gut microbes. Notably, *C.B* functioned as a gut microbiota-modulator effectively and restored gut microbial diversity and enhanced bacterial richness, demonstrating its capacity to ameliorate HFD-induced dysbiosis. Specifically, *C.B*-treated HFD mice exhibited significant enrichment of beneficial butyrate-producing bacterial taxa, including *Faecalibaculum* (genus), *Factobacillus* (genus) *and Erysipelotrichaceae* (family) (Figure 6). These SCFA-producing bacteria play a crucial role in maintaining intestinal epithelial barrier function through the production of SCFAs, attenuating systemic inflammatory responses and promoting metabolic homeostasis (Wang et al., 2023). Members of the *Lactobacillaceae* family have emerged as promising probiotic candidates with significant potential in promoting host metabolic health (Chuandong et al., 2024). Furthermore, *Erysipelotrichaceae* has been increasingly recognized for its anti-obesity properties and beneficial metabolic effects (Zeng et al., 2023; Tong et al., 2021). Furthermore, *C.B* intervention significantly reduced the relative abundance of *Desulfovibrionaceae*, a bacterial family associated with LPS production and pro-inflammatory responses (Zhao et al., 2020). Notably, *C.B* administration also attenuated the expansion of *Proteobacteria*, a well-established microbial marker of gut dysbiosis and intestinal barrier dysfunction (Shin et al., 2015). Importantly, some studies described that the gut microbiota of obese animals and humans exhibits a higher *Firmicutes*/*Bacteroidetes* ratio, proposing this ratio as an eventual biomarker. In the current study, HFD indeed significantly enhanced the ratio of *p_Firmicutes*/*p_Bacteroidetes*, while *C.B* administration had no effect on this ratio.

GDM is a major determinant of low-grade inflammation by disrupting the function of the intestinal barrier scaffold. Evidences showed GDM has strong association with metabolic disorder (Violi et al., 2023). Orally administered *C.B* spores germinate and grow in intestinal tracts, and produce large amounts of SCFAs (Sato and Tanaka, 1997). Several animal studies showed that LPS induced systematic inflammation and increased serum FFA level (Cani et al., 2008). Our findings demonstrate that *C.B* administration significantly reduced the abundance of LPS-producing *Desulfovibrionaceae*. Furthermore, the observed upregulation of tight junction protein-encoding genes, coupled with a marked decrease in circulating LPS levels, provides compelling evidence that *C.B* intervention effectively enhances intestinal barrier integrity and reduces endotoxemia. Importantly, *C.B* supplementation decreased the gut microbiota related to lipogenesis and increased the gut microbiota related to secondary bile acid biosynthesis (Figure 6E). These results were further supported by the decreased lipid content in plasma and in liver, however, the mechanism underlying this finding need further study.

Circulating levels of BCAA are related with IR and postulated as predictive biomarkers of T2DM (Zhou et al., 2019; White and Newgard, 2019; Ramzan et al., 2022). Our previous studies showed that BCAA supplementation elevated the plasma level of BCAA and counteracted exercise-attenuated IR in obese mice (Cao et al., 2024). These results prompted the importance of regulation the serum BCAA within a normal range. Bacteria are capable of numerous biochemical processes, including digestion of proteins or peptides that escaped absorption in the upper part of the digestive tract (Wang et al., 2022). It has been reported that gut bacterial proteins encompass a higher ratio of BCAA to other amino acids compared with the mammalian organism (Dai et al., 2011). Notably, the abundance of *B. stercoris* in human participants was positively correlate with BCAA (statistically significant for valine) and promotes NAFLD (Ni et al., 2023). Here, we found the top 10 KEGG pathways were enriched based on microbiota function. The up-regulated pathways of cecal microbiota in obese mice included lipid metabolism, glycan biosynthesis and metabolism and BCAA biosynthesis were significantly down regulated after 2-month *C.B* intervention. This was consistent with the decreases of serum BCAA/BCKA. It is widely accepted that some gut metabolites may leak into circulation, which may have directly impact on host metabolism (Stoeva et al., 2021; Wang et al., 2022). Therefore, we speculate the decreased serum levels of BCAA may partly result from the more tight junction in *C.B* treated HFD group mice. Altogether, the above results suggested that *C.B* can alleviate metabolic disorder of obese mice, at least partially through inhibition of LPS and BCAA biosynthesis.

BCAA levels in mouse blood are determined by the ingested food, tissue metabolism of BCAAs, and gut microbe (Kannaiyan et al., 2025). Gut bacteria possess autonomous BCAA biosynthesis capacity, and the entire BCAA biosynthetic pathway is induced in the gut microbiota of obese compared to lean humans (Ridaura et al., 2013). Meanwhile, it has been shown that the colonization of *Bacteroides* in obese mice enhances gut BCAA degradation efficiency, reducing microbial production of BCAA, ultimately leading to decreased circulating BCAA levels in the host (Yoshida et al., 2021). Another research found that increased BCAA biosynthesis and depletion for genes encoding the transport system for bacterial BCAA uptake (referred to as inward BCAA transport) in the gut microbiome of insulin-resistant individuals contribute to an increased BCAA pool (Pedersen et al., 2016). Therefore, it is possible that *C.B* affects the BCAA biosynthesis, degradation, and uptake by gut microbe. On the other hand, *C.B* may affect tissue BCAA metabolism in host. BCAAs cannot be synthesized *de novo* in mammalian cells. The plasma BCAA levels can be affected by BCAA uptake, catabolism, or release by tissue cells, which possibly affected by *C.B* through unknown mechanism. Nevertheless, the potential mechanisms by which *C.B* may influence BCAA levels guarantee further exploration.

In conclusion, our study revealed that orally administration of *Clostridium butyricum 337279* reversed obese mice metabolic disorder mainly by altering gut microbiota composition and functionality, which lowered circulating BCAA/BCKA, LPS levels, alleviate key obesity indexes (body weight, WAT, plasma TG/TC) and systematic inflammation. These results indicated a novel mechanism of *C.B* for obesity that improve metabolic disorder at least partially through decreasing LPS and BCAA production of intestinal flora. Our findings might contribute to further understanding of obesity and the development of innovative microbiome-based therapeutics.

## Data Availability

The datasets presented in this study can be found in online repositories. The names of the repository/repositories and accession number(s) can be found in the article/Supplementary material.

## Footnotes

^1^ https://www.graphpad.com/

## References

[ref1] Acciarino A. Diwakarla S. Handreck J. Bergola C. Sahakian L. McQuade R. M. (2024). The role of the gastrointestinal barrier in obesity-associated systemic inflammation. Obes. Rev. 25:e13673. doi: 10.1111/obr.13673, PMID: 38111141

[ref2] Cani P. D. Bibiloni R. Knauf C. Waget A. Neyrinck A. M. Delzenne N. M.. (2008). Changes in gut microbiota control metabolic endotoxemia-induced inflammation in high-fat diet-induced obesity and diabetes in mice. Diabetes 57, 1470–1481. doi: 10.2337/db07-1403, PMID: 18305141

[ref3] Cao W. Liu Y. Wei H. Dong Y. Sun H. Zhang X.. (2024). Aerobic exercise attenuates insulin resistance via restoring branched chain amino acids homeostasis in obese mice. Front. Nutr. 11:1451429. doi: 10.3389/fnut.2024.1451429, PMID: 39634544 PMC11615396

[ref4] Cassir N. Benamar S. La Scola B. (2016). Clostridium butyricum: from beneficial to a new emerging pathogen. Clin. Microbiol. Infect. 22, 37–45. doi: 10.1016/j.cmi.2015.10.014, PMID: 26493849

[ref5] Chuandong Z. Hu J. Li J. Wu Y. Wu C. Lai G.. (2024). Distribution and roles of Ligilactobacillus murinus in hosts. Microbiol. Res. 282:127648. doi: 10.1016/j.micres.2024.127648, PMID: 38367479

[ref6] Cresci G. A. Glueck B. McMullen M. R. Xin W. Allende D. Nagy L. E. (2017). Prophylactic tributyrin treatment mitigates chronic-binge ethanol-induced intestinal barrier and liver injury. J. Gastroenterol. Hepatol. 32, 1587–1597. doi: 10.1111/jgh.13731, PMID: 28087985 PMC5511097

[ref7] Dabke K. Hendrick G. Devkota S. (2019). The gut microbiome and metabolic syndrome. J. Clin. Invest. 129, 4050–4057. doi: 10.1172/JCI129194, PMID: 31573550 PMC6763239

[ref8] Dai Z. L. Wu G. Zhu W. Y. (2011). Amino acid metabolism in intestinal bacteria: links between gut ecology and host health. Front Biosci (Landmark Ed) 16, 1768–1786. doi: 10.2741/3820, PMID: 21196263

[ref9] Fan Z. Zhang X. Shang Y. Zou M. Zhou M. Qiukai E.. (2022). Intestinal Flora changes induced by a high-fat diet promote activation of primordial follicles through macrophage infiltration and inflammatory factor secretion in mouse ovaries. Int. J. Mol. Sci. 23:4797. doi: 10.3390/ijms23094797 35563189 PMC9100959

[ref10] Kang Y. Kang X. Yang H. Liu H. Yang X. Liu Q.. (2022). Lactobacillus acidophilus ameliorates obesity in mice through modulation of gut microbiota dysbiosis and intestinal permeability. Pharmacol. Res. 175:106020. doi: 10.1016/j.phrs.2021.106020, PMID: 34896249

[ref11] Kannaiyan S. P. Prasanna A. Chaganti S. Perin M. Dinakar S. Khedekar S.. (2025). Branched-chain amino acids in obesity and diabetes: implications and insights. Int J Med Biochem 8, 139–150. doi: 10.14744/ijmb.2025.16779

[ref12] Karusheva Y. Koessler T. Strassburger K. Markgraf D. Mastrototaro L. Jelenik T.. (2019). Short-term dietary reduction of branched-chain amino acids reduces meal-induced insulin secretion and modifies microbiome composition in type 2 diabetes: a randomized controlled crossover trial. Am. J. Clin. Nutr. 110, 1098–1107. doi: 10.1093/ajcn/nqz191, PMID: 31667519 PMC6821637

[ref13] Koh A. de Vadder F. Kovatcheva-Datchary P. Bäckhed F. (2016). From dietary Fiber to host physiology: short-chain fatty acids as key bacterial metabolites. Cell 165, 1332–1345. doi: 10.1016/j.cell.2016.05.041, PMID: 27259147

[ref14] Lee H. An J. Kim J. Choi D. Song Y. Lee C. K.. (2022). A novel bacterium, Butyricimonas virosa, preventing HFD-induced diabetes and metabolic disorders in mice via GLP-1 receptor. Front. Microbiol. 13:858192. doi: 10.3389/fmicb.2022.858192, PMID: 35655996 PMC9152154

[ref15] Lemieux I. Després J. P. (2020). Metabolic syndrome: past, present and future. Nutrients 12:3501. doi: 10.3390/nu12113501 33202550 PMC7696383

[ref16] Li Z. Zhang R. Mu H. Zhang W. Zeng J. Li H.. (2022). Oral Administration of Branched-Chain Amino Acids Attenuates Atherosclerosis by inhibiting the inflammatory response and regulating the gut microbiota in ApoE-deficient mice. Nutrients 14:5065. doi: 10.3390/nu14235065, PMID: 36501095 PMC9739883

[ref17] Ma L. Shen Q. Lyu W. lv L. Wang W. Yu M.. (2022). Clostridium butyricum and its derived extracellular vesicles modulate gut homeostasis and ameliorate acute experimental colitis. Microbiol Spectr 10:e0136822. doi: 10.1128/spectrum.01368-22, PMID: 35762770 PMC9431305

[ref18] Mann E. R. Lam Y. K. Uhlig H. H. (2024). Short-chain fatty acids: linking diet, the microbiome and immunity. Nat. Rev. Immunol. 24, 577–595. doi: 10.1038/s41577-024-01014-8, PMID: 38565643

[ref19] Mitev K. Taleski V. (2019). Association between the gut microbiota and obesity. Open Access Maced. J. Med. Sci. 7, 2050–2056. doi: 10.3889/oamjms.2019.586, PMID: 31406553 PMC6684436

[ref20] Nascimento J. C. Matheus V. A. Oliveira R. B. Tada S. F. S. Collares-Buzato C. B. (2021). High-fat diet induces disruption of the tight junction-mediated Paracellular barrier in the proximal small intestine before the onset of type 2 diabetes and Endotoxemia. Dig. Dis. Sci. 66, 3359–3374. doi: 10.1007/s10620-020-06664-x 33104937

[ref21] Neis E. P. Dejong C. H. Rensen S. S. (2015). The role of microbial amino acid metabolism in host metabolism. Nutrients 7, 2930–2946. doi: 10.3390/nu7042930, PMID: 25894657 PMC4425181

[ref22] Ni Y. Qian L. Siliceo S. L. Long X. Nychas E. Liu Y.. (2023). Resistant starch decreases intrahepatic triglycerides in patients with NAFLD via gut microbiome alterations. Cell Metab. 35, 1530–1547.e8. doi: 10.1016/j.cmet.2023.08.002, PMID: 37673036

[ref23] Oh S. F. Praveena T. Song H. Yoo J. S. Jung D. J. Erturk-Hasdemir D.. (2021). Host immunomodulatory lipids created by symbionts from dietary amino acids. Nature 600, 302–307. doi: 10.1038/s41586-021-04083-0, PMID: 34759313 PMC8999822

[ref24] Pedersen H. K. Gudmundsdottir V. Nielsen H. B. Hyotylainen T. Nielsen T. Jensen B. A.. (2016). Human gut microbes impact host serum metabolome and insulin sensitivity. Nature 535, 376–381. doi: 10.1038/nature18646, PMID: 27409811

[ref25] Potrykus M. Czaja-Stolc S. Stankiewicz M. Kaska Ł. Małgorzewicz S. (2021). Intestinal microbiota as a contributor to chronic inflammation and its potential modifications. Nutrients 13:3839. doi: 10.3390/nu13113839, PMID: 34836095 PMC8618457

[ref26] Ramzan I. Ardavani A. Vanweert F. Mellett A. Atherton P. J. Idris I. (2022). The association between circulating branched chain amino acids and the temporal risk of developing type 2 diabetes mellitus: a Systematic Review & Meta-Analysis. Nutrients 14:4411. doi: 10.3390/nu14204411, PMID: 36297095 PMC9610746

[ref27] Ren X. Xu J. Xu Y. Wang Q. Huang K. He X. (2023). Artemether attenuates gut barrier dysfunction and intestinal Flora imbalance in high-fat and high-fructose diet-fed mice. Nutrients 15:4860. doi: 10.3390/nu15234860, PMID: 38068719 PMC10707945

[ref28] Ridaura V. K. Faith J. J. Rey F. E. Cheng J. Duncan A. E. Kau A. L.. (2013). Gut microbiota from twins discordant for obesity modulate metabolism in mice. Science 341:1241214. doi: 10.1126/science.1241214, PMID: 24009397 PMC3829625

[ref29] Riedel S. Pheiffer C. Johnson R. Louw J. Muller C. J. (2021). Intestinal barrier function and immune homeostasis are missing links in obesity and type 2 diabetes development. Front Endocrinol (Lausanne) 12:833544. doi: 10.3389/fendo.2021.833544 35145486 PMC8821109

[ref30] Sato R. Tanaka M. (1997). Intestinal distribution and intraluminal localization of orally administered Clostridium butyricum in rats. Microbiol. Immunol. 41, 665–671. doi: 10.1111/j.1348-0421.1997.tb01909.x, PMID: 9343816

[ref31] Shin N. R. Whon T. W. Bae J. W. (2015). Proteobacteria: microbial signature of dysbiosis in gut microbiota. Trends Biotechnol. 33, 496–503. doi: 10.1016/j.tibtech.2015.06.011, PMID: 26210164

[ref32] Stoeva M. K. Garcia-So J. Justice N. Myers J. Tyagi S. Nemchek M.. (2021). Butyrate-producing human gut symbiont, Clostridium butyricum, and its role in health and disease. Gut Microbes 13, 1–28. doi: 10.1080/19490976.2021.1907272, PMID: 33874858 PMC8078720

[ref33] Tilg H. Zmora N. Adolph T. E. Elinav E. (2020). The intestinal microbiota fuelling metabolic inflammation. Nat. Rev. Immunol. 20, 40–54. doi: 10.1038/s41577-019-0198-4, PMID: 31388093

[ref34] Tong L. T. Xiao T. Wang L. Lu C. Liu L. Zhou X.. (2021). Plant protein reduces serum cholesterol levels in hypercholesterolemia hamsters by modulating the compositions of gut microbiota and metabolites. iScience 24:103435. doi: 10.1016/j.isci.2021.103435, PMID: 34927019 PMC8649741

[ref35] Violi F. Cammisotto V. Bartimoccia S. Pignatelli P. Carnevale R. Nocella C. (2023). Gut-derived low-grade endotoxaemia, atherothrombosis and cardiovascular disease. Nat. Rev. Cardiol. 20, 24–37. doi: 10.1038/s41569-022-00737-2, PMID: 35840742 PMC9284488

[ref36] Wang T. J. Larson M. G. Vasan R. S. Cheng S. Rhee E. P. McCabe E.. (2011). Metabolite profiles and the risk of developing diabetes. Nat. Med. 17, 448–453. doi: 10.1038/nm.2307, PMID: 21423183 PMC3126616

[ref37] Wang S. Li M. Lin H. Wang G. Xu Y. Zhao X.. (2022). Amino acids, microbiota-related metabolites, and the risk of incident diabetes among normoglycemic Chinese adults: findings from the 4C study. Cell Rep Med 3:100727. doi: 10.1016/j.xcrm.2022.100727, PMID: 35998626 PMC9512668

[ref38] Wang G. Liu J. Zhang Y. Xie J. Chen S. Shi Y.. (2023). Ginsenoside Rg3 enriches SCFA-producing commensal bacteria to confer protection against enteric viral infection via the cGAS-STING-type I IFN axis. ISME J. 17, 2426–2440. doi: 10.1038/s41396-023-01541-7, PMID: 37950067 PMC10689736

[ref39] Wang D. Wang L. Han L. Wang B. Shi R. Ye J.. (2023). Leucine-restricted diet ameliorates obesity-linked cognitive deficits: involvement of the microbiota-gut-brain Axis. J. Agric. Food Chem. 71, 9404–9418. doi: 10.1021/acs.jafc.3c01524, PMID: 37306277

[ref40] Wang L. Zhang P. Li C. Xu F. Chen J. (2022). A polysaccharide from Rosa roxburghii Tratt fruit attenuates high-fat diet-induced intestinal barrier dysfunction and inflammation in mice by modulating the gut microbiota. Food Funct. 13, 530–547. doi: 10.1039/D1FO03190B, PMID: 34932054

[ref41] White P. J. McGarrah R. W. Grimsrud P. A. Tso S. C. Yang W. H. Haldeman J. M.. (2018). The BCKDH kinase and phosphatase integrate BCAA and lipid metabolism via regulation of ATP-citrate Lyase. Cell Metab. 27, 1281–1293.e7. doi: 10.1016/j.cmet.2018.04.015, PMID: 29779826 PMC5990471

[ref42] White P. J. Newgard C. B. (2019). Branched-chain amino acids in disease. Science 363, 582–583. doi: 10.1126/science.aav0558, PMID: 30733403 PMC9940269

[ref43] Winer D. A. Luck H. Tsai S. Winer S. (2016). The intestinal immune system in obesity and insulin resistance. Cell Metab. 23, 413–426. doi: 10.1016/j.cmet.2016.01.003 26853748

[ref44] Wong J. M. de Souza R. Kendall C. W. Emam A. Jenkins D. J. (2006). Colonic health: fermentation and short chain fatty acids. J. Clin. Gastroenterol. 40, 235–243. doi: 10.1097/00004836-200603000-00015, PMID: 16633129

[ref45] Xu Z. Jiang W. Huang W. Lin Y. Chan F. K. Ng S. C. (2022). Gut microbiota in patients with obesity and metabolic disorders – a systematic review. Genes Nutr. 17:2. doi: 10.1186/s12263-021-00703-6 35093025 PMC8903526

[ref46] Yao Y. Cai X. Fei W. Ye Y. Zhao M. Zheng C. (2022). The role of short-chain fatty acids in immunity, inflammation and metabolism. Crit. Rev. Food Sci. Nutr. 62, 1–12. doi: 10.1080/10408398.2020.1854675 33261516

[ref47] Yoshida N. Yamashita T. Osone T. Hosooka T. Shinohara M. Kitahama S.. (2021). Bacteroides spp. promotes branched-chain amino acid catabolism in brown fat and inhibits obesity. iScience 24:103342. doi: 10.1016/j.isci.2021.103342, PMID: 34805797 PMC8586802

[ref48] Zeng H. He S. Xiong Z. Su J. Wang Y. Zheng B.. (2023). Gut microbiota-metabolic axis insight into the hyperlipidemic effect of lotus seed resistant starch in hyperlipidemic mice. Carbohydr. Polym. 314:120939. doi: 10.1016/j.carbpol.2023.120939, PMID: 37173019

[ref49] Zhao Z. Chen L. Zhao Y. Wang C. Duan C. Yang G.. (2020). Lactobacillus plantarum NA136 ameliorates nonalcoholic fatty liver disease by modulating gut microbiota, improving intestinal barrier integrity, and attenuating inflammation. Appl. Microbiol. Biotechnol. 104, 5273–5282. doi: 10.1007/s00253-020-10633-9, PMID: 32335723

[ref50] Zhou M. Shao J. Wu C. Y. Shu L. Dong W. Liu Y.. (2019). Targeting BCAA catabolism to treat obesity-associated insulin resistance. Diabetes 68, 1730–1746. doi: 10.2337/db18-0927, PMID: 31167878 PMC6702639

