# Supplementary figures and images for: Clostridium Butyricum 337279 shapes the gut microbiota to attenuate metabolic disorder in diet-induced obese mice
Source: Front Microbiol. 2025 May 9;16:1580847. doi: 10.3389/fmicb.2025.1580847 (PMC12098643; doi:10.3389/fmicb.2025.1580847)

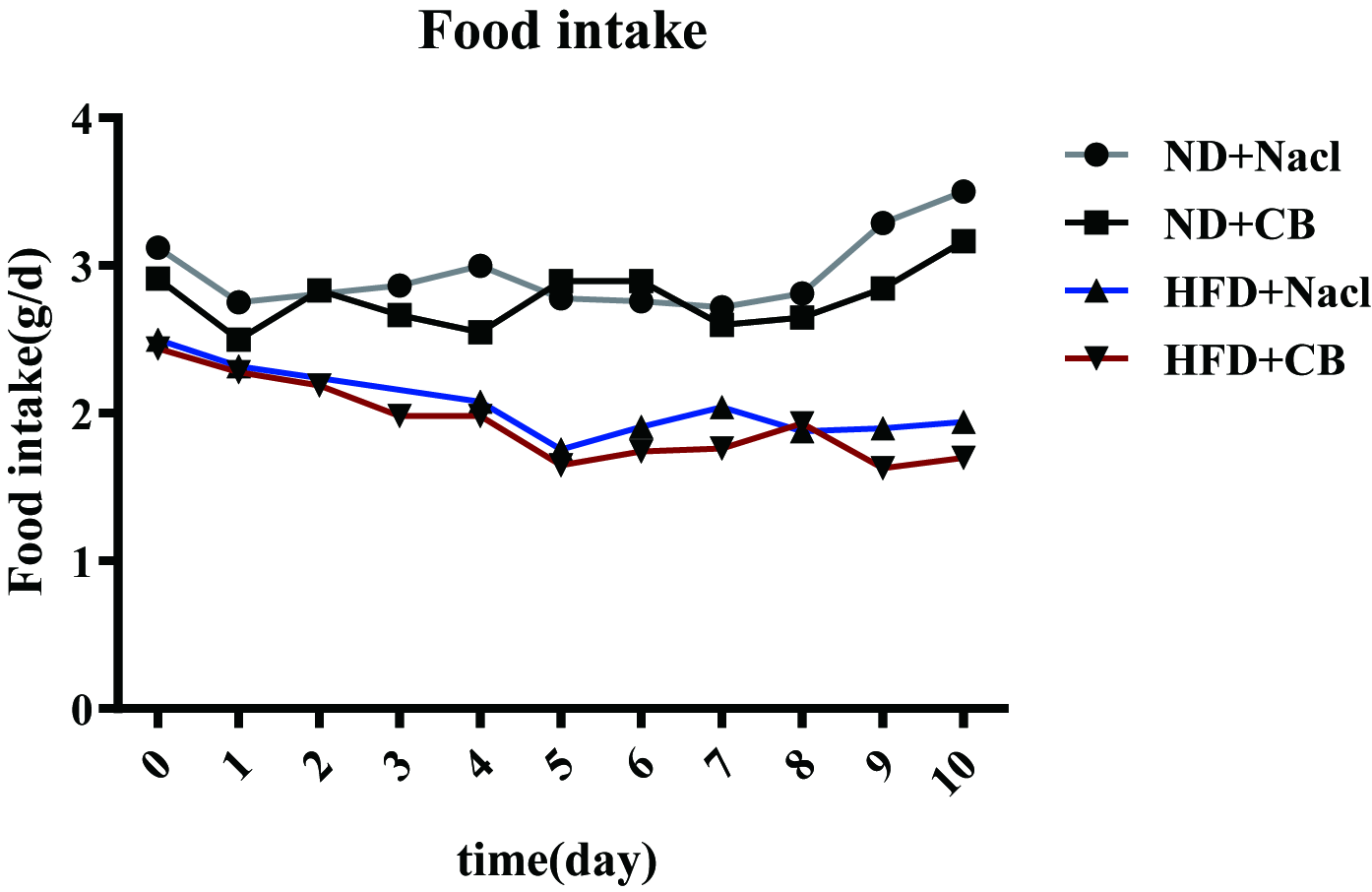

Supplement: Supplementary file 1 [file Image_1.tif]
